# The Influence of Knee Extensor and Ankle Plantar Flexor Strength on Single-Leg Standing Balance in Older Women

**DOI:** 10.3390/jfmk8020067

**Published:** 2023-05-18

**Authors:** Weerasak Tapanya, Sinthuporn Maharan, Patchareeya Amput, Noppharath Sangkarit, Boonsita Suwannakul

**Affiliations:** 1 Department of Physical Therapy, School of Allied Health Sciences, University of Phayao, Phayao 56000, Thailand; sinthuporn.ma@up.ac.th (S.M.); patchareeya.am@up.ac.th (P.A.); noppharath.sa@up.ac.th (N.S.); boonsita.sa@up.ac.th (B.S.); 2 Unit of Excellence of Human Performance and Rehabilitations, University of Phayao, Phayao 56000, Thailand

**Keywords:** maximum voluntary isometric contraction, older adult, sarcopenia, fall, weakness

## Abstract

Impaired balance is a significant risk factor for falls among older adults. The precise impact of lower-extremity muscles, including the proportion of muscle strength, on the performance of single-leg standing balance tests in older individuals is very interesting. The aim of this study is to examine the correlation between the knee extensor (KE), ankle plantar flexor (AP) muscle strength, and performance in single-leg standing balance tests in older females. Additionally, it aims to evaluate the combined proportion of KE and AP muscle strength in maintaining balance during single-leg standing. A total of 90 older females (mean age 67.83 ± 8.00 years) were recruited. All participants underwent maximum voluntary isometric contraction (MVIC) testing of the KE and AP muscles, as well as single-leg standing balance tests with eyes open (SSEO) and eyes closed (SSEC). To examine the influence of KE and AP muscle strength on balance performance, multiple regression analysis was conducted. Low correlations were found between SSEO and MVIC of KE and AP muscles, but moderate correlations were found with percentage of MVIC to body weight ratio (%MVIC/BW). The best model for SSEO included 0.99 times of the %MVIC/BW of AP and 0.66 times that of KE muscles as independent predictor variables (r = 0.682). In conclusion, AP muscle strength was found to have a greater impact on single-leg standing balance compared with KE muscle strength.

## 1. Introduction

Falls are a significant public health concern for older adults worldwide, including in Thailand. Falls represent a significant public health concern for the aging population, with approximately one-third of individuals over 65 experiencing such incidents every year. Unfortunately, falls occur on an annuasl basis and are the second most frequent cause of unintentional injury-related deaths, which is a matter of great concern [1]. The prevalence of falls among Thai older adults was high, with over 25% of participants reporting at least one fall in the previous year [2]. The prevalence of falls increased with age, and females had a higher prevalence of falls than males [3]. Falls are a significant problem among older individuals and can result in various physical, psychological, and social problems, including injuries, fear of falling, reduced mobility, depression and anxiety, social isolation, and increased healthcare costs [4,5,6,7]. Comprehending how muscle strength affects balance during a single stance can aid in designing targeted and more effective fall prevention interventions.

The progressive loss of skeletal muscle mass and function that accompanies aging, known as sarcopenia, is a widely recognized consequence of aging in humans. Sarcopenia affects both muscle quantity and quality and is characterized by reductions in muscle mass, strength, and power, which can lead to a decline in functional performance and an increased risk of falls and fractures in older adults [8,9]. The pathophysiology of sarcopenia is complex and multifactorial, involving a combination of intrinsic factors such as changes in hormonal and metabolic regulation, mitochondrial dysfunction, and inflammation [10], as well as extrinsic factors such as physical inactivity, poor nutrition, and chronic diseases [11]. The decline in muscle mass, strength, and power associated with sarcopenia can result in impaired postural stability, decreased gait speed, and reduced ability to perform activities of daily living [11,12,13,14]. In addition, sarcopenia can also affect the sensory and motor systems involved in maintaining balance, such as changes in proprioception and reaction time [15], further exacerbating the risk of falls.

In the event of a balance loss, it is crucial to rapidly halt the body’s momentum and keep the center of mass within the limits of the base of support. During single-leg standing, the knee and ankle muscles work together to maintain balance [16]. Knee extensor (KE) and ankle plantar flexor (AP) muscle strengths are important for standing balance as they are responsible for maintaining upright posture and pushing the body forward and upward [17,18,19,20]. In contrast, knee flexor and ankle dorsiflexor muscles play a secondary role in maintaining balance, with their contribution being relatively small [19,21]. The KE muscles, including the quadriceps, work to extend the knee joint and prevent it from collapsing [22], while the AP muscles, including the gastrocnemius and soleus, work to maintain ankle stability and prevent the foot from rolling inward or outward [17]. These muscles work in a coordinated manner to make small adjustments in body position and maintain the center of mass within the base of support. When the body shifts out of balance, the knee and ankle muscles contract to reposition the body and maintain stability [23]. However, the specific influence of these muscles, such as the proportion of muscle strength, on the performance of single-leg standing balance tests in older individuals is not well established. 

Hill et al., 2021, investigated the relationship between muscle thickness and echo intensity of the KE and AP muscles with postural sway, mobility, and physical function in older adults [24]. They found that there were significant correlations between the muscle thickness and echo intensity of the vastus lateralis and gastrocnemius medialis with Timed-Up and Go-test (TUGT) (r = −0.432 to −0.492) and five times sit-to-stand (FTSST) (r = −0.473 to −0.596) performance [24]. Moreover, Lopez et al., 2017; Rech et al., 2014; and Wilhelm et al., 2014 suggests that these measures are negatively correlated with TUGT and 30 s sit-to-stand tests (r = −0.49 to −0.56) [25,26,27]. Muscle thickness is an indicator of muscle size and is associated with muscle strength. Therefore, a negative correlation indicates that individuals with greater muscle thickness have better performance on these functional tests of mobility and lower-body strength. Previous studies have investigated the association between balance and muscle strength, focusing on individual muscle groups rather than considering the proportion of muscle work that influences the ability to maintain balance. Therefore, there is a need for research that examines the specific contribution of individual muscle groups, as well as their co-working, to the ability to maintain balance in older adults.

A well-established fact is that the ankle muscles play a crucial role in reducing postural sway during static standing. Additionally, the plantar flexors have been identified as crucial agonist muscles for minimizing postural sway in the anteroposterior direction [17]. Most of the previous studies have primarily focused on assessing the strength of either the KE or the AP muscles to maintain balance. Understanding the co-activation patterns of the AP and KE muscles may provide insight into the mechanisms underlying balance control in older adults. Moreover, identifying the specific proportions of co-activation that are associated with better balance performance could inform the development of targeted exercise interventions for improving balance in this population. Therefore, the purpose of this study is to investigate the relationship between the KE and AP muscle strength and single-leg standing balance test performance in older females. In addition, it is crucial to consider the combined proportion of muscle strength influence of both the KE and AP muscles as they work synergistically to maintain balance. This highlights the importance of examining lower-extremity muscle strength as a collective factor rather than focusing solely on one muscle group when studying the impact on balance.

## 2. Materials and Methods

### 2.1. Study Population

The study included 90 older females living in Muang District, Phayao Province, Thailand. The recruitment of participants was carried out through community leaders and a network of village health officers. The sample size was determined using G*Power version 3.1.5 based on the bivariate normal model correlation from the study conducted by Yoshizawa and Yoshida [28] with an effect size of 0.76. An alpha level of 0.05 and a power factor of 80% were considered in the determination. The baseline data of the subjects are presented in Table 1. The inclusion criteria for the study required participants to be older, healthy females or have a controllable chronic illness, such as diabetes or hypertension, and be able to walk independently. The exclusion criteria included lower-extremity musculoskeletal problems, such as osteoarthritis and rheumatoid arthritis, broken or dislocated bones, or neurological issues affecting muscle strength and balance, such as stroke, spinal cord disease, and Parkinson’s disease, as well as communication, vision, and hearing issues.

### 2.2. Data Collection

The researcher fully explained the study’s purpose and data collection procedure to the participants. Prior to participation, participants signed a consent form. Basic personal information such as age, body weight, height, and BMI was collected. Participants were given five minutes to practice submaximal isometric contractions of the KE and AP muscles against an assessor force before the trial began. After they became familiar with the movements, the experiment commenced. At least five minutes of rest were provided between each test trial, and two trials of maximum voluntary isometric contraction (MVIC) of the KE and AP muscles and single-leg standing balance test were measured. The variable measurement sequence and followed method were as per the following.

#### 2.2.1. The Maximum Voluntary Isometric Contractions (MVIC) Test of the KE Muscle

**Figure 1:**
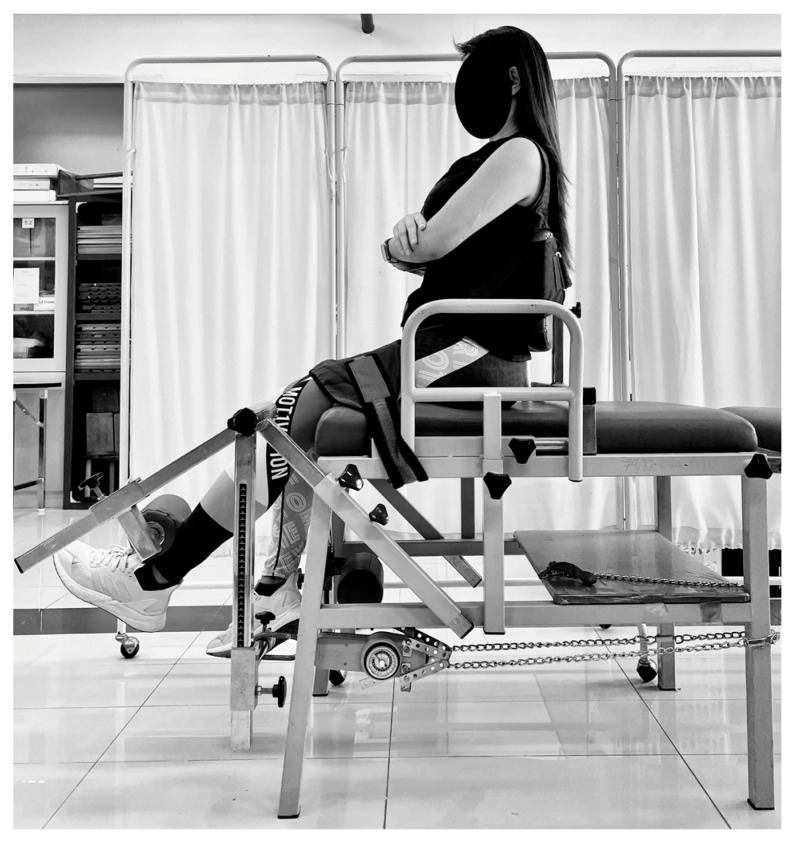
Maximum voluntary contractions (MVCs) test using the push–pull dynamometer that was mounted to the NK table’s leg.

The seating of the NK table was adjusted to accommodate the participants’ leg lengths, and their knees were flexed to a 60-degree angle. The trunk and upper legs were secured with a safety belt, while the push–pull dynamometer (Baseline^®^ Analog Hydraulic Push–Pull Dynamometer, Fabrication Enterprises Inc. (FEI) based in Elmsford, New York, NY, USA) was positioned 1 cm above the lateral malleolus, as depicted in Figure 1. Participants were instructed to extend their knee as far as possible against the push–pull dynamometer, hold the contraction for 4 s, and perform three testing trials with a two-minute rest interval between each trial. The maximum force obtained was recorded, and the MVCs tests were quantified in kilograms. Although the dynamometer can measure force in Newtons, the measurements provided by push–pull dynamometer models in this study were in kilograms or pounds. The specific unit of measurement depends on the model and configuration being used.

#### 2.2.2. The Maximum Voluntary Isometric Contractions (MVIC) Test of the AP Muscle

**Figure 2:**
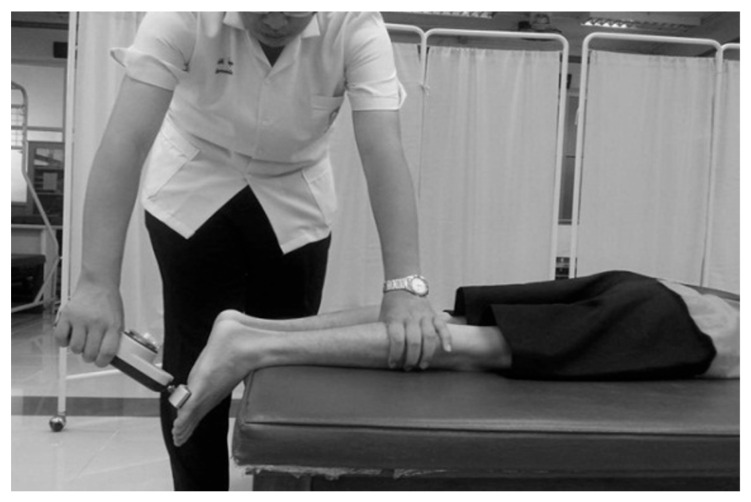
Push–pull dynamometer measurement of the maximum contraction force of the ankle plantar flexor muscle.

Before commencing the AP muscle test, the participants were instructed to stretch their calf muscles. They were then positioned prone on the bed with their feet extended over the edge, and the push pads of the push–pull dynamometer (Baseline^®^ Analog Hydraulic Push–Pull Dynamometer, United States) were adjusted and placed on the ball of the foot to be measured, as shown in Figure 2. To ensure the participants were familiar with the test, they were allowed to perform one trial of submaximal contraction. The researcher then instructed the participants to push their toes against the push pads of the dynamometer with maximal force and sustain the contraction for 4 s [29]. The test consisted of three trials, with a two-minute rest interval between each trial. The maximum contraction force of the AP muscle was determined as the highest value obtained from the three trials and was quantified in kilograms.

#### 2.2.3. Single-Leg Standing Balance Test

The participants’ dominant legs were assessed through a self-reported questionnaire or interview, in which they were asked to identify the leg they would naturally use to perform tasks of kicking a ball. The single-leg standing balance test was initiated with participants standing with both feet on the ground and looking straight ahead. The researcher then instructed the participants to shift their weight to their dominant leg while crossing their arms in front of their chest. The non-dominant knee was flexed at a 90-degree angle, and a timer was started to measure the duration of balance maintained by the participants. The test was conducted for a total duration of 120 s, and the timer was immediately stopped when either the opposite foot touched the ground or the hands left the chest. The test was repeated three times, with a two-minute break between each trial. The average of testing time in seconds of the three trials was recorded by the assessor. The single-leg standing balance test was conducted under two conditions, eyes open (SSEO) and eyes closed (SSEC).

### 2.3. Statistical Analysis

The normality of the data distribution was assessed using the skewness, kurtosis, and Shapiro–Wilk test. All variables had a normal distribution except SSEO and SSEC variables for which the distribution can be considered normal after considering the skewness and kurtosis values. Descriptive statistics were used to describe the characteristics of the participants. The Pearson’s product moment correlation coefficient was used to determine the correlation between the maximum KE and AP contraction force and the time taken to complete the SSEO and SSEC tests. To determine the strength of the relationship between the two variables based on the correlation coefficient, the following scale was used in this study: a correlation coefficient of 0.9 to 1 was considered a strong relationship, 0.5 to 0.9 was considered a moderate association, 0.2 to 0.5 was considered a weak association, and 0 to 0.2 was considered no relationship [30]. To investigate the proportion of KE and AP muscle strength that influenced balance performance, a multiple regression analysis was performed using the stepwise multiple linear regression analysis technique. All statistical analyses were conducted using SPSS version 21 (SPSS Inc., Chicago, IL, USA), and a significance level of 0.05 was applied to all statistical tests.

### 2.4. Ethical Approval

The study was approved by the Human Research Ethics Committee of the University of Phayao (No. 2/168/60) and was conducted in accordance with the ethical principles of the Declaration of Helsinki.

## 3. Results

The MVIC and percentage of MVIC to body weight ratio (%MVIC/BW) of KE and AP muscles, as well as the average duration of the single-leg standing balance test, are presented in Table 2.

**Figure 3:**
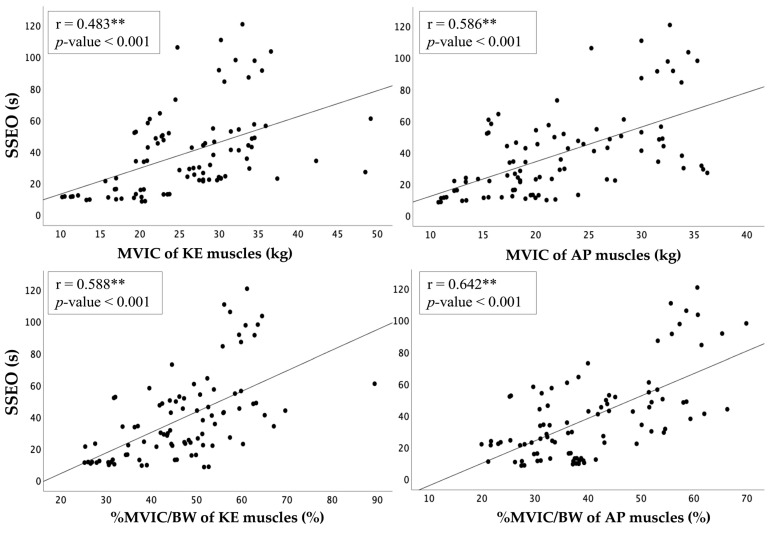
Correlation between MVIC or %MVIC/BW of KE and AP muscles and single-leg standing balance performance. Note: ** Correlation is significant at *p* < 0.001.

Of the demographic information of the participants, only the age was analyzed for correlation with the single-leg standing balance test at low levels (r = −0.262 to −0.303, *p* < 0.05). A weak to moderate correlation was observed between SSEO and the MVIC of KE and AP muscles (r = 0.483 to 0.586, respectively; *p* < 0.001), while a moderate correlation was found between SSEO and the %MVIC/BW of KE and AP muscles (r = 0.588 to 0.642, respectively; *p* < 0.001). Conversely, SSEC was found to be weakly correlated with both MVIC and %MVIC/BW of KE and AP muscles (r = 0.308 to 0.424, *p* < 0.01). Table 3 and Figure 3 depict the correlation findings.

The multiple regression analysis yielded two equation models for single-leg standing performance. **Model 1** indicated that only one variable, 1.41 times the %MVIC/BW of AP muscles, had a significant effect on SSEO (r = 0.642, *p* < 0.05), with the R^2^ for the equation indicating that approximately 40.5% of the variance could be accounted for. **Model 2**, the best model for SSEO (*p* < 0.001), incorporated 0.99 times the %MVIC/BW of AP muscles and 0.66 times the %MVIC/BW of KE muscles as independent predictor variables (r = 0.682), with the R^2^ for the equation indicating that roughly 45.2% of the variance could be explained as shown in Table 4.

## 4. Discussion

As people age, they tend to lose muscle mass, which can lead to a decrease in strength and function. This loss of muscle mass and function can have significant consequences for balance and mobility [31]. Sarcopenia is a condition characterized by the loss of muscle mass and function that occurs with aging [31]. As muscle mass decreases, the muscles that support the body and enable movement become weaker. This can affect a person’s ability to maintain their balance, particularly when standing or walking [31]. Without sufficient muscle strength, a person may be more prone to falls, which can lead to serious injuries, particularly in older adults.

There have been several studies exploring the correlation between demographic data and single-leg standing balance performance in older individuals. Some of the demographic factors that have been investigated include age, sex, body mass index (BMI), and medical conditions. In this study, we analyzed the demographic data of the participants, using only the age to determine their correlation with single-leg standing balance test scores at low levels as r = −0.262 to −0.303 (*p* < 0.05). Although the results of the study showed that demographic data were associated with a low level of balance performance, it should be noted that these variables may have an impact on an individual’s ability to maintain balance. Some studies have found that sex is not a significant predictor of balance performance [32], while others have found that males tend to have better balance control than females [33]. The study by Boonwang et al. revealed a significant difference in sway velocity between males and females in the older adult Thai population during right leg standing with eyes open conditions [34]. Specifically, females had a higher sway velocity than males [34]. Moreover, the study indicated that males demonstrated better stability as evidenced by lower sway velocity during the right leg standing balance tests [34]. Consequently, it was deemed appropriate to conduct a separate analysis of older adult females in this study. Moreover, there is a well-established correlation between age and single-leg standing balance performance in older individuals [32,35]. As people age, they typically experience a decline in balance control due to changes in various physiological systems, such as the vestibular, visual, and somatosensory systems [36,37]. This decline in balance control can increase the risk of falls and injury in older individuals. These findings suggest that age is an important factor to consider when assessing balance control in older individuals, consistent with previous studies [32,33]. Moreover, no correlation was found between body height and balance performance. Some studies found no significant association between body height and single-leg standing balance performance in a sample of older individuals with and without fall risk [38].

There is a significant low correlation between KE muscle strength (r = 0.308 to 0.483), AP strength (r = 0.416 to 0.586), and single-leg balance in the older adult population. This result has shown that decreased KE and AP muscle strength is associated with poor balance, which can increase the risk of falls and related injuries in older adults. The KE muscles are responsible for generating the force required to stabilize the knee joint during standing on one leg [22], while the APs generate the force required to maintain ankle joint stability and control the movement of the body’s center of mass [17]. Therefore, both groups of muscles play an important role in controlling the movement of the COM, which can affect balance [23]. Upon analysis of the muscle strength to body weight ratio, it was found that there is a stronger correlation with the ability to stabilize in SSEO (r = 0.588 to 0.642) compared with the ankle and knee muscle strength alone. This is because the AP and KE strength to body weight ratio takes into account the individual’s body weight, which can affect their ability to maintain balance [39]. Previous studies have shown that obese individuals exhibit significantly lower knee flexor and extensor strength compared with those with normal weight when data are normalized per body weight. The study suggests that normalization per body weight, rather than the more commonly used normalization per fat-free mass, can better represent the load bearing on muscles, which is a major biomechanical constraint in individuals [39]. Therefore, these findings suggest that the muscle strength to body weight ratio is an important factor to consider when assessing the relationship between AP/KE strength and single-leg balance in older individuals.

The primary aim of this study was to determine the relative contribution of muscle strength in the AP and KE muscles, working together to maintain balance in older adults. This study sought to build upon previous research that has primarily focused on the relationship between balance ability and each of the lower limbs’ muscle strength. By using multiple regression analyzing the proportional importance of these specific muscle groups, this study provided valuable insight into the mechanisms underlying balance control in older adults. The results of the multiple regression analysis indicated that Model 1 showed a significant influence of only %MVIC/BW of AP muscles on SSEO balance ability with a beta value of 1.411. The beta value represents the amount of change in SSEO balance ability for every one-unit change in %MVIC/BW of AP muscles while holding all other independent variables constant. The findings of Model 2 revealed that the %MVIC/BW of AP muscles and %MVIC/BW of KE muscles had a significant impact on SSEO, with betas of 0.988 and 0.659, respectively. The results also showed that the influence of lower-limb strength on SSEO was distributed unequally, with two-thirds of the impact coming from AP muscle strength, while KE muscle strength contributed one-third of the influence. AP muscles and KE muscles were co-working together to maintain single-leg balance control in older individuals. During the single-leg stance, the APs were primarily responsible for controlling the movement of the body’s center of mass in the anterior–posterior direction [17,40,41]. A previous study has shown that the first muscle synergy can primarily be attributed to an ankle control strategy, while the second muscle synergy is associated with a knee control strategy [16]. The result of this study found that three major muscle synergies were involved in the single-limb stance, two of which were ankle-dominant, and one was knee-dominant. While both the AP strength and the KE strength are important for maintaining single-leg balance in older individuals, this study suggests that the AP strength may be more important. The biomechanical explanation for this finding is that the APs are more directly involved in controlling the movement of the body’s center of mass during the single-leg stance, whereas the KEs play a more supportive role in this process [16]. When the body experiences a balance perturbation, such as an unexpected push or slip, the ankle muscles are among the initial muscles to be activated in order to restore balance [42]. This is because the ankle joint is the primary joint involved in maintaining balance during standing and walking. AP muscles play a critical role in the initial response to perturbations. In addition, studies have found that individuals with stronger ankle muscles are better able to recover from balance perturbations and maintain balance [43]. When maintaining the single-leg balance stance, the KE muscles play an important role in stabilizing the knee joint by producing an isometric contraction that locks the joint in a stable position. This is often referred to as the “knee locking” mechanism, which occurs when the knee joint is fully extended, and the KE muscles, particularly the quadriceps muscles, contract to hold the joint in place. Once the knee joint is locked, the KE muscles require only a small amount of activation to maintain this position during the single-leg balance stance. This is because the locked knee joint is inherently stable, requiring minimal muscular effort to maintain its position.

However, it should be noted that there are some limitations to this study. The SSEO and SSEC measures used to assess balance are functional tests that evaluate the time a subject can maintain balance, but they do not provide information on the displacement of the body’s center of mass (CoM), which represents true balance. While these measures are widely used and accepted due to their ease of administration and minimal equipment requirements, future research could benefit from the inclusion of gold standard tests, such as body CoM measurements using force platform instruments, to provide a more comprehensive evaluation of balance. Furthermore, while this study focused on measuring muscle strength using MVIC, it would also be valuable to examine muscle activity using electromyography (EMG) to provide insight into the muscle activation patterns and coordination during balance tasks. This would offer a more complete understanding of the mechanisms underlying the relationship between muscle strength and single-leg balance in the older adult population. This study provided valuable insights into the relationship between the AP and KE muscle strength and single-leg balance in older adults; future research could benefit from the inclusion of additional measures, such as body CoM displacement and muscle activity using EMG, to further enhance our understanding of the underlying mechanisms of balance control in this population. Another limitation of this study is that it did not perform a separate analysis of correlations and regression for male participants, despite the well-documented dissimilarities in strength development patterns between the sexes. To advance the knowledge of this area, future research should consider conducting separate correlation and regression analyses for males to validate the results observed in older females. This research gap can be addressed to achieve a more comprehensive understanding of the factors that influence strength development in both males and females.

## 5. Conclusions

In conclusion, the muscle strength to body weight ratio plays a crucial role in the relationship between the AP/KE strength and single-leg balance in older individuals. Moreover, this study suggested that the influence of lower-limb strength on SSEO was distributed unequally; the AP muscle strength had a significantly greater impact on single-leg standing balance compared with the KE muscle strength. This key result underscores the crucial role of the AP muscle strength in maintaining balance and highlights the importance of targeted exercises for this muscle group in promoting balance and preventing falls.

## Figures and Tables

**Table 1:** Subject demographic data and anthropometric characteristics variables (*n* = 90).

Variables	Mean ± SD
Age (years)	67.83 ± 8.00
Body weight (kg)	55.95 ± 10.44
Height (cm)	154.48 ± 7.30
Body mass index; BMI (kg/m^2^)	23.44 ± 3.94

**Table 2:** Muscle strength and single-leg standing balance performance variables (*n* = 90).

Variables	Mean ± SD
MVIC of KE muscles (kg)	25.76 ± 7.94
MVIC of AP muscles (kg)	22.17 ± 7.20
%MVIC/BW of KE muscles	46.69 ± 12.25
%MVIC/BW of AP muscles	40.51 ± 12.24
Single-leg standing balance test with eye open; SSEO (s)	38.82 ± 26.92
Single-leg standing balance test with eye closed; SSEC (s)	5.11 ± 4.92

**Table 3:** Correlation between demographic information of the participant, knee extensor, ankle plantar flexor muscle strength, and single-leg standing balance performance.

		SSEO	SSEC
**Demographic data** **Variables**	Age	−0.262 * (*p* value = 0.013)	−0.303 ** (*p* value = 0.004)
	Weight	0.049 (*p* value = 0.648)	0.101 (*p* value = 0.122)
	Height	0.040 (*p* value = 0.711)	0.050 (*p* value = 0.643)
	BMI	0.039 (*p* value = 0.713)	0.112 (*p* value = 0.294)
**MVIC of KE muscles**		0.483 ** (*p* value < 0.001)	0.308 ** (*p* value < 0.001)
**MVIC of AP muscles**		0.586 ** (*p* value < 0.001)	0.416 ** (*p* value < 0.001)
**%MVIC/BW of KE muscles**		0.588 ** (*p* value < 0.001)	0.321 ** (*p* value < 0.001)
**%MVIC/BW of AP muscles**		0.642 ** (*p* value < 0.001)	0.424 ** (*p* value < 0.001)

Note: ** Correlation is significant at *p* < 0.01. * Correlation is significant at *p* < 0.05. SSEO = single-leg standing balance test with eye open, SSEC = single-leg standing balance test with eye closed, MVIC = maximum voluntary isometric contraction, %MVIC/BW = percentage of MVIC to body weight ratio, KE = knee extensor, and AP = ankle plantar flexor.

**Table 4:** Model of regression analysis for single-leg standing balance performance with different predictive variables.

Model	Included Variables	*Β*	*p* Value	r	Adjusted r^2^	SEE
1	Constant	−18.352	0.018 *	0.642	0.405	20.77
	%MVIC/BW of AP muscles	1.411	<0.001 **			
2	Constant	−32.010	<0.001 **	0.682	0.452	19.92
	%MVIC/BW of AP muscles	0.988	<0.001 **			
	%MVIC/BW of KE muscles	0.659	0.004 *			

Note: ** Correlation is significant at *p* < 0.001. * Correlation is significant at *p* < 0.005. SEE is standard error of estimation.

## Data Availability

Data are unavailable due to ethical restrictions.
